# Amplification of LTRs of extrachromosomal linear DNAs (ALE-seq) identifies two active *Oryco* LTR retrotransposons in the rice cultivar Dongjin

**DOI:** 10.1186/s13100-022-00274-2

**Published:** 2022-06-13

**Authors:** Hyunjin Koo, Soomin Kim, Hyun-Seung Park, Sang-Ji Lee, Nam-Chon Paek, Jungnam Cho, Tae-Jin Yang

**Affiliations:** 1 Department of Agriculture, Forestry and Bioresources, Plant Genomics and Breeding Institute, College of Agriculture and Life Sciences, Seoul National University, 1 Gwanak-ro, Gwanak-gu, 08826 Seoul, Republic of Korea; 2 CAS-JIC Centre of Excellence for Plant and Microbial Science, 200032 Shanghai, China

**Keywords:** Long terminal repeat retrotransposon, Transposition, ALE-seq, *Oryza sativa*, Dongjin

## Abstract

Long terminal repeat retrotransposons (LTR-RTs) make up a considerable portion of plant genomes. New insertions of these active LTR-RTs modify gene structures and functions and play an important role in genome evolution. Therefore, identifying active forms of LTR-RTs could uncover the effects of these elements in plants. Extrachromosomal linear DNA (eclDNA) forms during LTR-RT replication; therefore, amplification LTRs of eclDNAs followed by sequencing (ALE-seq) uncover the current transpositional potential of the LTR-RTs. The ALE-seq protocol was validated by identification of *Tos17* in callus of Nipponbare cultivar. Here, we identified two active LTR-RTs belonging to the *Oryco* family on chromosomes 6 and 9 in rice cultivar Dongjin callus based on the ALE-seq technology. Each *Oryco* family member has paired LTRs with identical sequences and internal domain regions. Comparison of the two LTR-RTs revealed 97% sequence identity in their internal domains and 65% sequence identity in their LTRs. These two putatively active *Oryco* LTR-RT family members could be used to expand our knowledge of retrotransposition mechanisms and the effects of LTR-RTs on the rice genome.

**Supplementary Information:**

The online version contains supplementary material available at 10.1186/s13100-022-00274-2.

## Background

Transposable elements (TEs) are mobile DNA sequences that contribute to the size variation and evolution of all eukaryotic genomes, especially plant genomes. Most plant species have a wide range of TEs, which make up more than 85% of the genome in plants with large genomes such as wheat (*Triticum aestivum*) and barley (*Hordeum vulgare*) [1–4]. TEs increase their copy numbers and insert into new regions of the genome. The transposition of TEs can induce structural rearrangements, generating mutations and affecting genome stability. In addition, these movements affect gene expression and function, ultimately influencing evolution and genome adaptation [5].

TEs are divided into two classes: retrotransposons (class I), which mobilize via a copy-and-paste mechanism; and DNA transposons (class II), which transpose via a cut-and-paste mechanism [6]. Long terminal repeat retrotransposons (LTR-RTs) are ubiquitous class I retrotransposons that are interspersed in all eukaryote genomes [7]. In most plants, LTR-RTs contribute to genome size expansion via their rapid proliferation [8, 9]. For example, LTR-RTs account for more than 70% of the maize (*Zea mays*) and wheat (*Triticum aestivum*) genomes [8, 10]. Intact LTR-RTs contain two similar or identical LTRs that flank an internal region containing a functional domain, target site duplications (TSDs), a polypurine tract (PPT), and a primer-binding site (PBS) [6]. Most LTR-RTs are classified into two superfamilies, *Copia* and *Gypsy*, which differ in their sequences and the composition of the internal conserved domains [6, 11]The *Gypsy* superfamily is the most highly represented superfamily in most plant genomes [12–14].

Since transposition of LTR-RTs is a major driving force for genome evolution, various studies have aimed to identify active LTR-RTs [15–18]. The transposition cycle of LTR-RTs begins with the transcription of genomic copies, followed by reverse transcription of the LTR-RT transcripts to form cDNA, also known as extrachromosomal linear DNA (eclDNA). These eclDNAs integrate into the nucleus and influence strand transfer [19]. Therefore, although the production of eclDNAs does not necessarily represent transposition events directly, it is considered a strong indication of transposition potential [20]. The transpositional potential of LTR-RTs has been revealed by identifying eclDNA via amplification of LTRs of eclDNAs followed by sequencing (ALE-seq) [15].

Rice (*Oryza sativa*) is an important cereal crop, providing a major source of food worldwide [21]. 40% of the rice genome consists of most known types of TEs, including LTR-RTs and DNA transposons [22]. Among these, LTR-RTs make up ~ 22% of the rice genome [23]. ALE-seq analysis was successfully used to detect eclDNAs, such as heat stress–activated *Go-on* and activated *Tos17*, in *O. sativa* ssp. *japonica* cv. Nipponbare callus [15]. Although active LTR-RTs have been identified in this model rice variety, little is known about active transposition in non-reference rice varieties. To close this gap, we conducted ALE-seq analysis to identify the transpositionally competent LTR-RTs during callus culture in the non-reference Korean rice variety Dongjin. To facilitate the identification of putatively active LTR-RTs, we interrogated the rice genome to search for structurally intact LTR-RTs, which would likely possess strong mobility. Importantly, ALE-seq detected eclDNAs derived from two *Oryco* family LTR-RTs, which are unique in Dongjin callus. Our work unveils previously unknown mobile DNAs in a non-reference rice genome and will help broaden the breadth of our knowledge on crop genome plasticity and stability.

## Results

### Identification and annotation of intact LTR-RTs

We tried to detect extrachromosomal linear DNA (eclDNA) forms of active LTR-RTs in Korean rice cultivar Dongjin by ALE-seq. The transpositionally competent LTR-RT should contain an intact structure, including two identical LTRs, a PBS, a PPT, a TSD, and conserved internal domains. Taking all these criteria into consideration, we discovered intact LTR-RTs using several LTR-RTs detection tools and custom python scripts (Additional file 2: Fig S1). We identified 1,783 intact LTR-RTs, including 1,226 *Gypsy*-type (68.8%) and 557 *Copia-*type (31.2%) LTR-RTs, in the *O. sativa* reference genome sequence. The *Del* family as well as the *Tat* family were prominent in the *Gypsy* superfamily, and the *Tork* family was the most abundant in the *Copia* superfamily (Additional file 1: Table S1-3).

Phylogenetic analysis of the LTR-RTs based on their reverse transcriptase (RT) domain distinguished the *Copia* and *Gypsy* superfamilies. The *Copia* superfamily was grouped into the *Oryco*, *Tork*, *Retrofit*, and *Sire* families; the *Gypsy* superfamily was grouped into the *Tat*, *Del*, *Reina*, and *Crm* families (Additional file 2: Fig S2). The *Tat* family accounted for the largest number of *Gypsy* superfamily members and had more variants than the other families. We plotted the pattern of full-length LTR-RTs on the phylogenetic tree to examine the length distribution of the LTR-RTs across the entire family (Additional file 2: Fig S3). A comparison of the LTRs on the left versus the right side for each element revealed that most elements were active during the past one million years (Myr), except for the *Sire* family (about 1–2 Myr ago) (Additional file 1: Table S4, Additional file 2: Fig S4).

### Identification of putatively active LTR-RTs in Dongjin callus

**Fig. 1:**
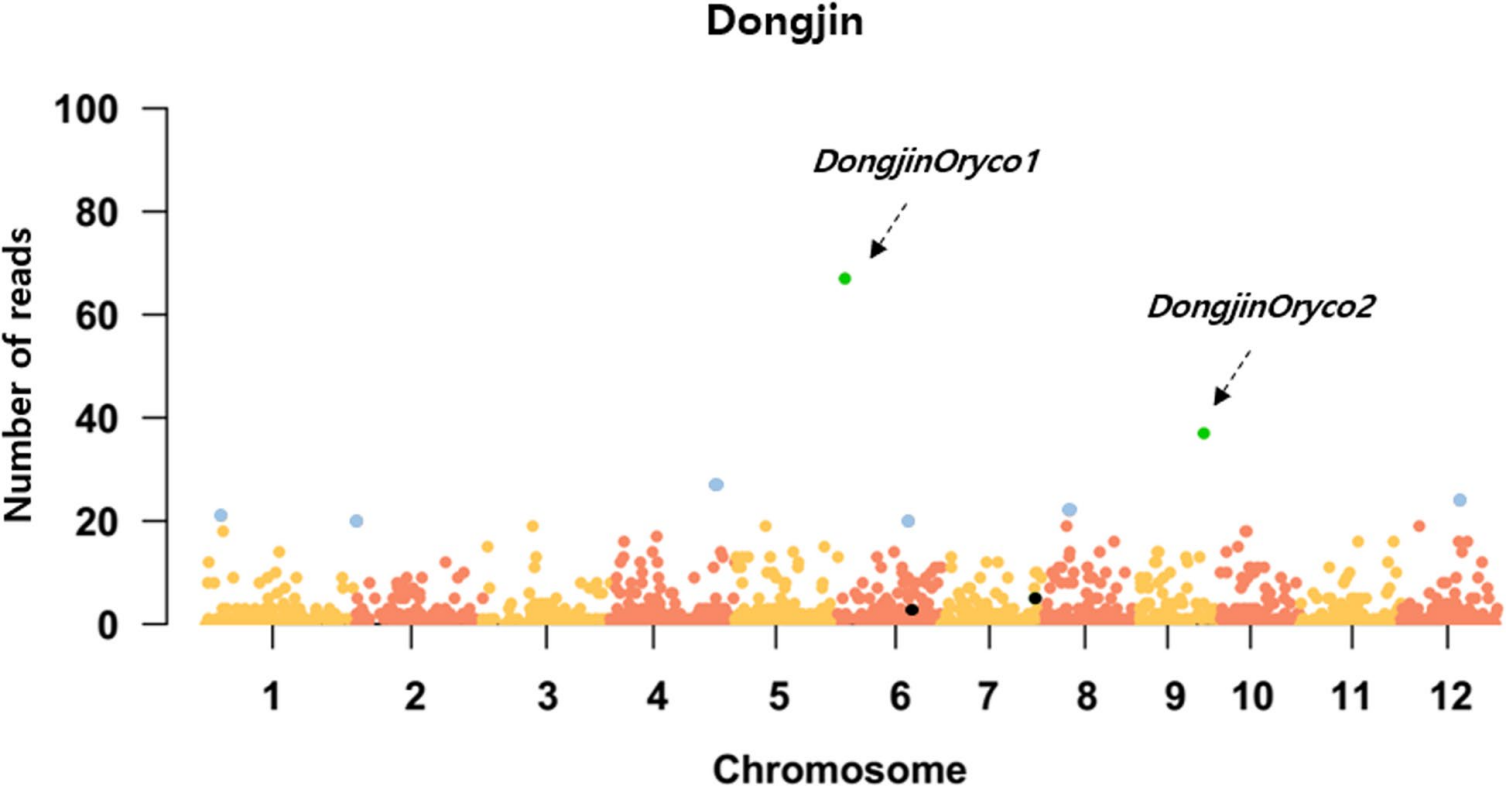
**Manhattan plot of ALE-seq reads from Dongjin rice callus.** Each dot represents a normalized value for reads mapped to each LTR-RT. Green dots represent LTR-RTs with strong transpositional activity. Blue dots represent LTR-RTs with moderate transpositional activity. Black dots indicate *Tos17* (Chr7) and *Tos19* (Chr6), which are known to have transpositional potential in Nipponbare callus [15]

**Fig. 2:**
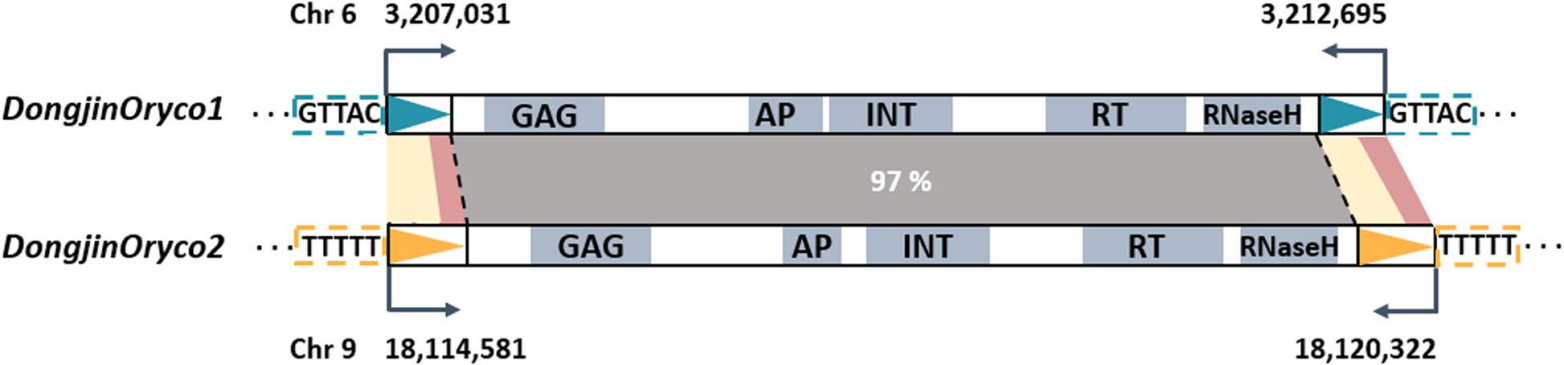
**Structural characteristics of the two active LTR-RTs.** Green and yellow triangles indicate LTR regions. Green and yellow dashed boxes indicate TSDs. Gray boxes indicate the internal domains of LTR-RTs: GAG: capsid protein, AP: aspartic protease, INT: integrase, RT: reverse-transcriptase; TSD: target site duplication. Gray panel represents DNA sequence similarity of the internal region between the two active LTR-RTs. The pale yellow panels and pink panels between the LTR sequences of the two active LTR-RTs indicate sequence similarities of 61% and 92%, respectively

The ALE-seq method was recently developed to identify candidate active LTR-RTs that have retrotranspositional activity and thus produce eclDNAs. Two LTR-RTs, *Tos17* and *Tos19*, which are known to be active in rice, were successfully detected as active LTR-RTs by ALE-seq using *O. sativa* cultivar Nipponbare callus [15]. In this study, we conducted ALE-seq to identify active LTR-RTs in callus of another rice variety, Dongjin. Mapping of ALE-seq reads from Dongjin callus identified signatures of eclDNA production derived from two LTR-RTs located on chromosomes 6 and 9 (Fig. 1). These putatively active LTR-RTs belong to the *Oryco* family and were therefore named *DongjinOryco1* and *DongjinOryco2* (Fig. 2, Additional file 1: Table S2, Additional file 2: Fig S5).

**Fig. 3:**
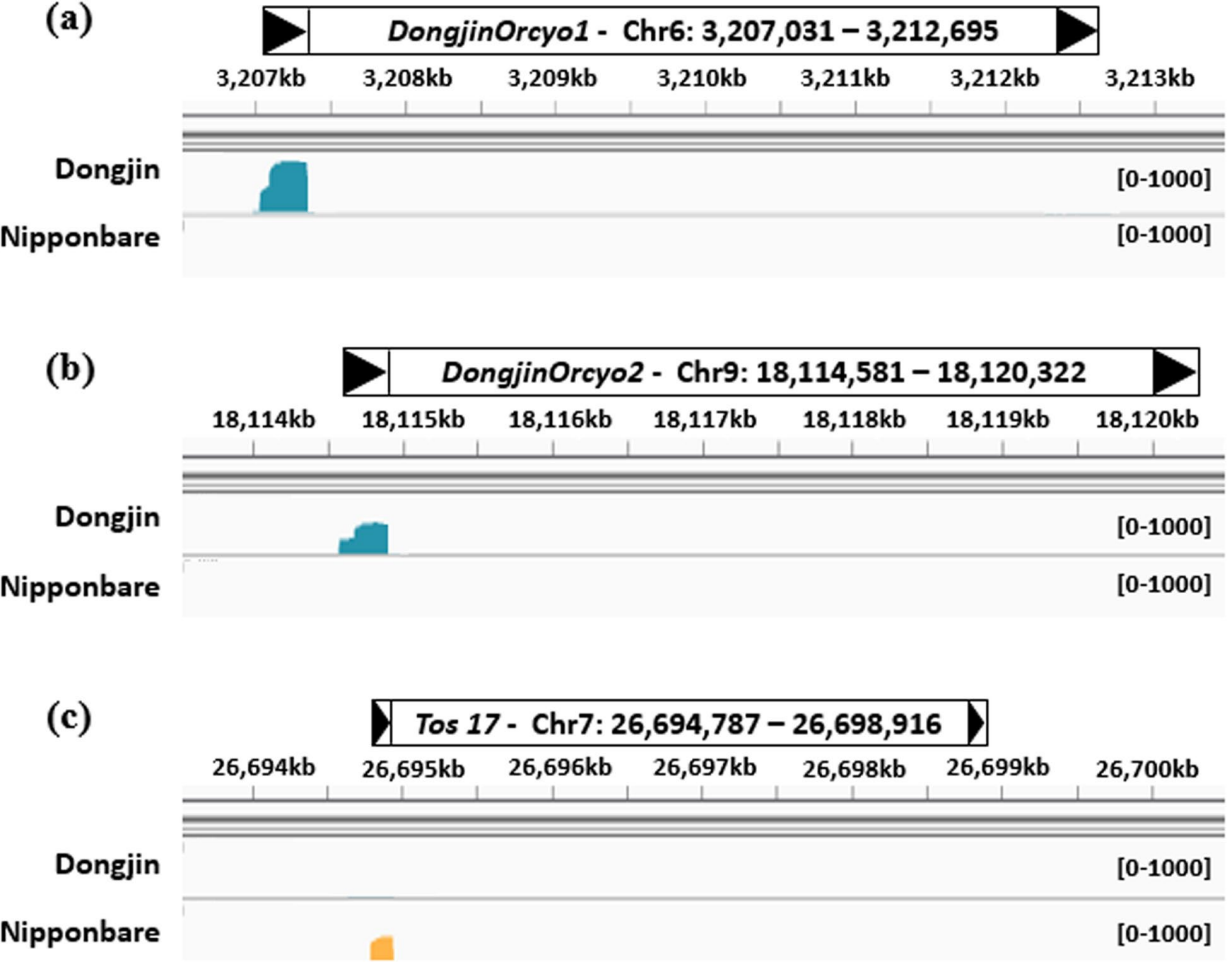
**Mapping of ALE-seq reads on the pseudochromosomes of the Nipponbare reference genome.** Read coverage plots and the positions of active LTR-RTs in Dongjin (**a, b**) and Nipponbare [15] (**c**). The genome browser images show the number of reads mapped to the chromosomal positions on the 5′ LTRs and primer binding sites of the three LTR-RTs

BLAST searches revealed that *DongjinOryco1* and *DongjinOryco2* are transcribed in various tissues, such as shoots, roots, and flowers, in diverse rice accessions (Additional file 1: Table S5-6). However, *DongjinOryco1* and *DongjinOryco2* were not detected in the callus of Nipponbare rice using ALE-seq. The ALE-seq reads were uniquely mapped to 5′ LTR regions and primer binding sites of the internal domains of the active LTR-RTs *DongjinOryco1* and *DongjinOryco2* in Dongjin callus (Fig. 3a and b) and *Tos17* in Nipponbare callus (Fig. 3c), respectively. The other six *Tat* family members showed moderate transpositional potential, and their map positions were identified on chromosomes 1, 2, 4, 6, 8, and 12 (Fig. 1, Additional file 1: Table S2).

### Structural characteristics of *DongjinOryco1* and *DongjinOryco2*

The putative active *Oryco* elements *DongjinOryco1* and *DongjinOryco2* have intact structures, including 100% identical sequences between the left and right LTR structures (Additional file 2: Fig S6) and the conserved internal domain region (GAG, AP, INT, RT, and RNaseH) (Additional file 2: Fig S7). The *Oryco* elements share 97% identical conserved internal sequences. However, the LTR sequences show diversity between *DongjinOryco1* and *DongjinOryco2*, with 61% and 92% sequence similarity for the 5′ and 3′ regions of the LTRs, respectively (Fig. 2, Additional file 2: Fig S5). Six other *Tat* family members showed relatively high (moderate) ALE-seq read depth, although the depth was much lower than that of the two *Oryco* family members. The *Tat* family members shared 59–96% sequence similarity for the entire internal region and 97.73–100% sequence similarity between the left and right LTR pair of each element (Additional file 1: Table S7, Additional file 2: Fig S7-9).

**Fig. 4:**
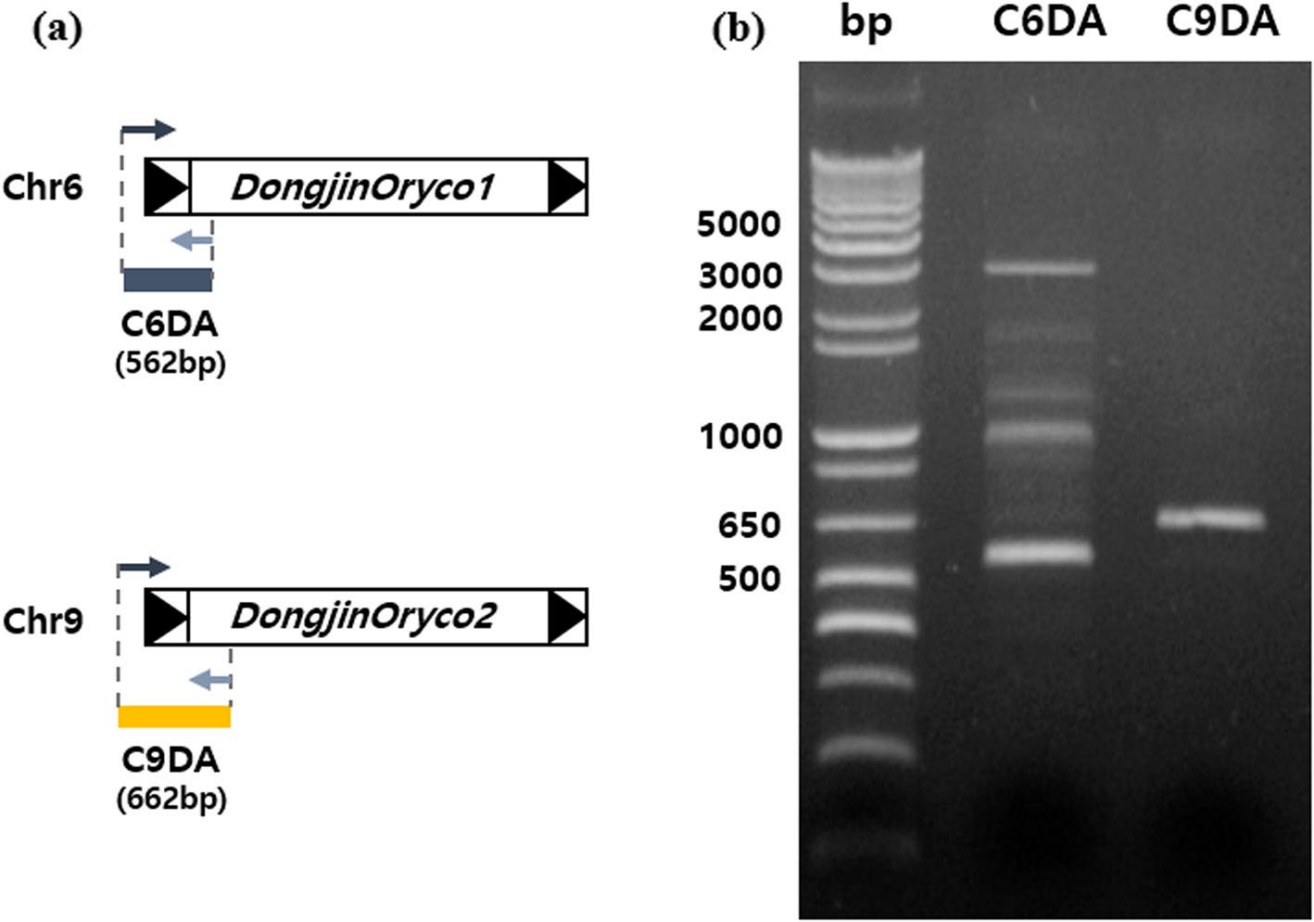
**Validation of the chromosomal positions of active LTR-RTs in Dongjin callus.** (a) Primer design based on the Nipponbare reference genome position to validate the co-localization of LTR-RTs in Dongjin callus. (b) PCR and gel electrophoresis of the two targets

We annotated and identified the intact LTR-RTs using the Nipponbare reference genome. Since the positions of LTR-RTs can differ among varieties, we validated the chromosomal positions of *DongjinOryco1* and *DongjinOryco2* by PCR amplification using primers designed based on the flanking site and internal LTR-RTs located on chromosomes 6 and 9 (Fig. 4).

## Discussion

### Retrotranspositional activity and structural integrity of the active LTR-RTs

ALE-seq analysis discovered a total of eight potentially active LTR-RTs in Dongjin callus including two *Oryco* family members and six *Tat* family members. The putative transposition time and retrotranscriptional activity of an LTR-RT can be estimated by comparing its LTR sequences [24] and the conservation of its internal domain regions [25]. The two *Oryco* family members were more conserved than the six *Tat* family members in terms of their overall structure, suggesting that the two *Oryco* LTR-RTs transposed recently and maintain higher transpositional potential than the six *Tat* family members.

*Gypsy* elements are more abundant in rice than *Copia* elements, which is similar to findings for *Solanum*, *Populus*, and *Capsicum* species [12–14]. *Copia* elements are usually more transcriptionally active than *Gypsy* elements [26, 27]. Elements that are abundant throughout the genome are more likely to be silenced by the host genome, whereas elements that are less abundant in the genome reduce the efficiency of the host’s silencing mechanisms [26–28]. *Tat* elements belonging to the *Gypsy* superfamily and *Oryco* elements belonging to the *Copia* superfamily accounted for 28.49% and 4.77% of the rice LTR-RTs, respectively (Additional file 1: Table S1). Therefore, the strong retrotranspositional potential of the *Oryco* elements belonging to the *Copia* superfamily appears to be supported by their strong structural integrity as well as their lower copy numbers in the rice genome [15].

### ALE-seq identifies new active LTR-RTs in Dongjin callus

LTR-RTs are transcriptionally activated when plants are exposed to environmental stress, such as heat, cold, salt, or tissue culture conditions [15, 18, 29–32]. However, it is difficult to directly confirm the transpositional potential of an LTR-RT due to post-transcriptional control [33]. The ALE-seq method was recently introduced to identify the direct retrotransposition potential of an LTR-RT by detection of eclDNA. Using this method, *Tos17*, which is known to be a major factor in tissue culture–induced mutations, was detected as having transpositional activity in the callus of *O. sativa* variety Nipponbare [15]. The *Tos17* system has greatly contributed to functional genomics studies of rice. However, this technique has been limited to Nipponbare, which was selected as a reference cultivar among japonica rice accessions [34]. Because functional genomics tools are also needed for non-reference varieties, we performed ALE-seq analysis using the callus of Korean rice variety Dongjin.

Notably, *Tos17* was highly active in Nipponbare callus but not in Dongjin callus (Fig. 3). *Tos17* elements are activated in most rice varieties, but not in the Moritawase variety, which has higher DNA methylation levels than other varieties, although the *Tos17* sequence in Moritawase is identical to that of Nipponbare [35]. Epigenetic variation might induce the silencing of *Tos17* in the Moritawase variety [35]. Similarly, the *Oryco* family members showed high transpositional potential in Dongjin callus but not in Nipponbare callus, which may have been influenced by the various epigenetic or genetic mutations that exist between the Dongjin and Nipponbare varieties.

TE amplification generates abundant genetic diversity in domesticated cultivars of rice and many other crops [1, 36–40]. The Dongjin and Nipponbare varieties exhibit large genetic variations [41]. Here, we identified two LTR-RTs, *DongjinOryco1* and *DongjinOryco2*, which are active in Dongjin callus but not in Nipponbare using ALE-seq method. These results suggest that the differential activation of diverse LTR-RTs might have influenced the diversification of rice varieties, which will require further investigation. In conclusion, ALE-seq is a robust approach to unearth hidden mobile DNAs and can be applicable to other plant species, varieties, and tissues to dissect the genome dynamics and plasticity.

## Methods

### Identification and characterization of LTR-RTs

*De novo* annotation of LTR-RTs in the rice reference genome (MSU7.0; http://rice.plantbiology.msu.edu) was performed with LTRharvest using the following options: minlenltr = 100, maxlenltr = 5000, and similar = 80. The plant-specific tRNAs used to screen PBS regions were obtained from plantRNA (http://seve.ibmp.unistra.fr/plantrna/). To filter out strictly intact LTR-RTs, we also scanned for the presence of tRNA and internal domains using LTRdigest with the following options: pptlen = c[8, 40], pbsalilen = c[8, 40], and pbsoffset = c(0,10). The flanking 5-bp regions at the start and end of each LTR-RT with more than 60% sequence homology were considered to be TSDs. Conserved domains (such as gag and pol proteins) were investigated using the TEsorter tool, which uses the gydb database containing GAG, AP, INT, RT, and RNaseH domain sequences [42] (Additional file 2: Fig S1).

To confirm the relatedness of the annotated LTR-RT families, RT amino acid sequences for each family were aligned using MAFFT [43]. The phylogenetic tree was constructed using the aligned RT amino acid sequences based on the maximum-likelihood method via IQ-TREE [44].

The pair of LTR sequences was aligned with MAFFT, and sequence divergence (*k*) between the pair of LTRs was estimated using Kimura’s two-parameter mo*Del* [45]. The insertion ages of the LTR-RTs were estimated using the formula T = *k*/2*r* (*r*: substitution rate of 1.3 × 10^−8^ substitutions per site per year) [46].

### Plant materials and ALE-seq analysis

Rice callus was generated from mature embryos of *O. sativa* ssp. *japonica* cv. Dongjin seeds. Dehulled seeds were sterilized with 2% sodium hypochlorite for 30 min and washed three times in sterile water. The sterilized seeds were inoculated on N6D medium containing 2 mg/L 2,4-D and cultured under continuous light at 30 °C for 2 weeks [47].

Total genomic DNA was extracted from the callus using a GeneAll Plant SV Midi Kit (GeneAll Biotechnology Co. Ltd., Seoul, Korea) following the manufacturer’s protocol. Genomic DNA (300 ng) was used to generate the ALE-seq library. The first step in ALE-seq was to connect an adapter containing the T7 promoter sequence to the end of 5’ end of the eclDNA, followed by *in vitro* transcription using T7 RNA polymerase. Reverse transcription was then performed using a primer that binds to the transcript of the PBS region, and LTR-RTs showing transposition potential were extracted [15]. The ALE-seq library was analyzed on the Illumina MiSeq platform with 30-bp paired-end reads by Macrogen (Seoul, Korea). The raw ALE-seq data for *O. sativa* ssp. *japonica* cv. Dongjin were deposited in the National Center for Biotechnological Information (NCBI) Sequence Read Archive (SRA, http://www.ncbi.nlm.nih.gov/sra) (accession number: SRR17128798).

The adapter sequences and poor-quality reads were filtered from the raw reads using Trimmomatic-0.33 [48]. Paired-end sequences were mapped to the rice reference genome (MSU7.0) using Bowtie2 (-X 3,000) [49]. The number of reads mapped throughout each LTR-RT was counted with the FeatureCounts tool using the customized annotation file generated by *de novo* LTR-RT prediction pipelines [50]. The IGV genome browser was used to visualize the read depth pattern [51].

### Validation of active LTR-RTs in Dongjin rice

The hit positions were manually inspected to identify the complete structures of active LTR-RTs. PCR verification was conducted to confirm that the active LTR-RTs in Dongjin were present at the same location in the Dongjin genome as in the reference genome. PCR primer pairs were designed using Primer 3 (http://bioinfo.ut.ee/primer3-0.4.0/). Forward primers were located in the flanking region of LTR-RTs, whereas reverse primers were located in the internal parts of LTR-RTs. The PCR products were separated on 1% agarose gels (Additional file 1: Table S8).

BLAST searches were conducted for the putatively active LTR-RTs to examine their transcriptional activity in various tissues or cultivars. BLASTN analysis was performed using the two candidate LTR-RTs as queries against the expressed sequence tag (EST) database using the parameter settings E-value < e^− 5^ and identity > 95% (https://blast.ncbi.nlm.nih.gov/Blast.cgi).

## Supplementary Information

**Additional file 1.**

**Additional file 2.**

## Data Availability

Raw read ALE-seq data are publicly available in the NCBI sequence read archive under accession number SRR17128798.

## Abbreviations

TE: transposable element
LTR-RT: long terminal repeat retrotransposon
eclDNA: extrachromosomal linear DNA
ALE-seq: amplification of LTRs of eclDNAs followed by sequencing
EST: expressed sequence tag
