# Supplementary material for: Amplification of LTRs of extrachromosomal linear DNAs (ALE-seq) identifies two active Oryco LTR retrotransposons in the rice cultivar Dongjin
Source: Mob DNA. 2022 Jun 13;13:18. doi: 10.1186/s13100-022-00274-2 (PMC9190103; doi:10.1186/s13100-022-00274-2)
Supplement: Supplementary file 2 — Additional file 2. [file 13100_2022_274_MOESM2_ESM.docx]

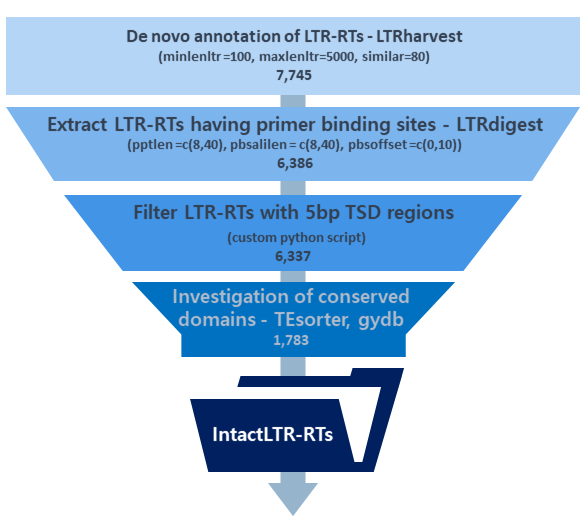


**Fig S1.** Pipeline for LTR-RT identification in the *O. sativa* genome (MSU7 version).


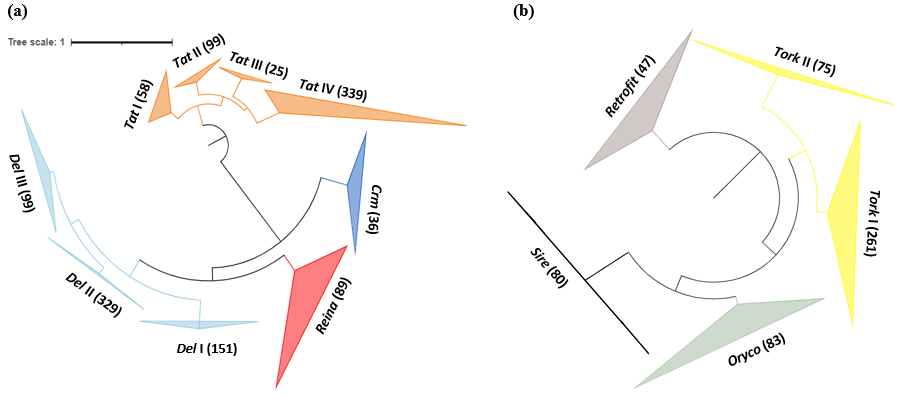
 **Fig S2.** Phylogenetic analysis of active long terminal repeat retrotransposons (LTR-RTs) based on the reverse-transcriptase (RT) domain. Maximum likelihood phylogenies of **(a)** *Gypsy* and **(b)** *Copia* superfamily members. In the tree, branch color denotes each family of LTR-RTs. Numbers indicate the number of members in each family. The amino acid sequences of the RT domains were aligned using MAFFT.


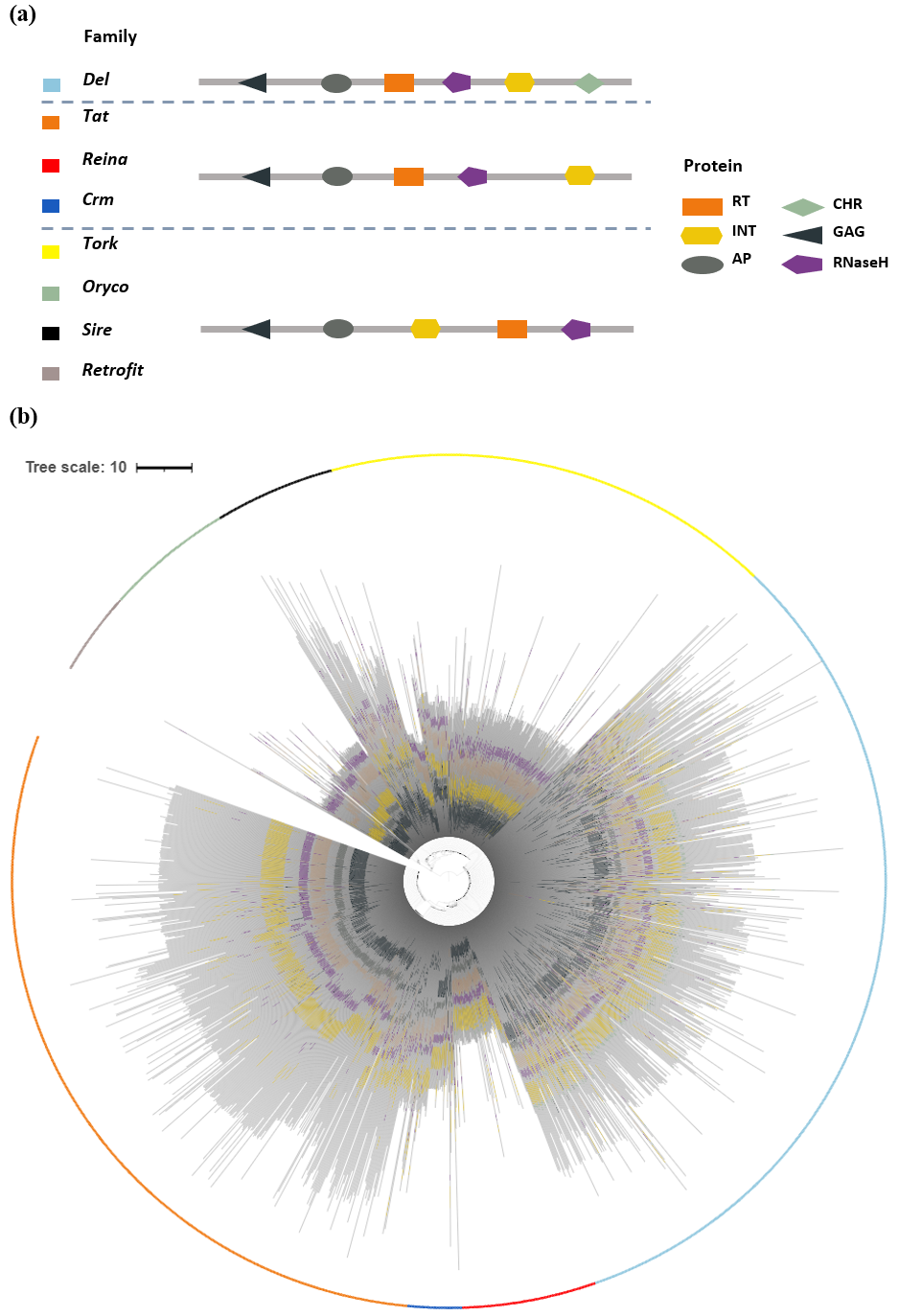


**Fig S3.** Domain structures and phylogenetic analysis of LTR-RT family members **(a)** Representative domain structures in each family. **(b)** Phylogenetic tree constructed using reverse-transcriptase (RT) domain sequences based on the maximum-likelihood method. Black triangle represents the GAG domain; gray circle represents the AP domain; orange rectangle represents the RT domain; yellow hexagon represents the INT domain; green diamond represents the CHR domain; purple pentagon represents the RNaseH domain. In the tree, the colors represent each LTR-RT family. Gray line indicates each amino acid sequence length in the LTR-RTs.


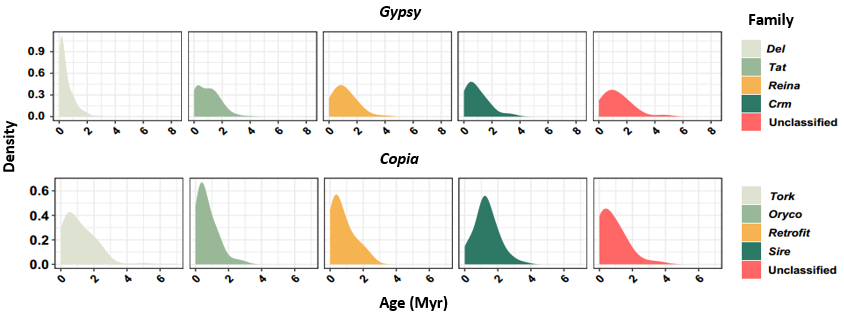


**Fig S4.** Age distribution of LTR-RTs for each family.


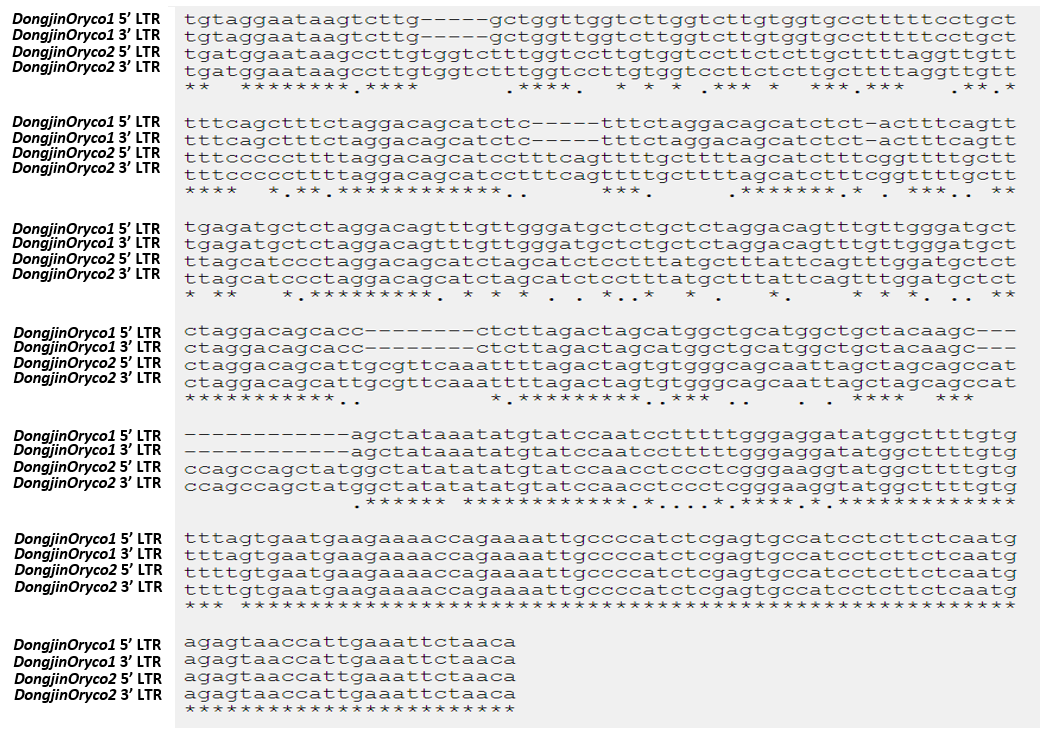
 **Fig S5.** Nucleotide sequence alignment of the LTR sequences of the two *putative* active LTR-RTs.


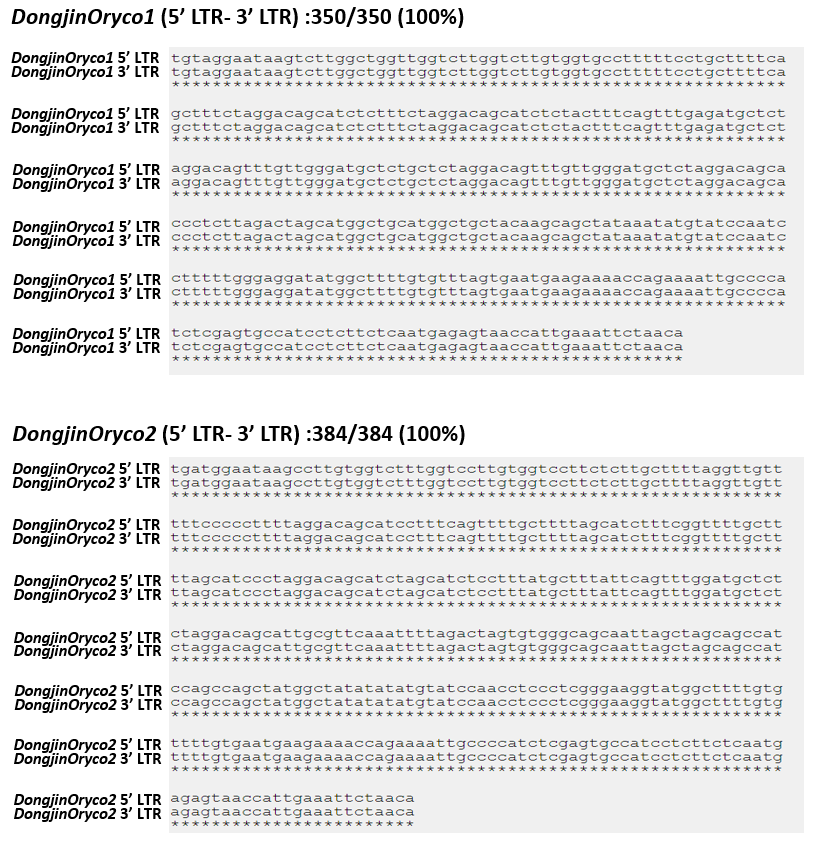
 **Fig S6.** Nucleotide sequence alignment of LTR pairs for each active LTR-RT belonging to the *Oryco* family.


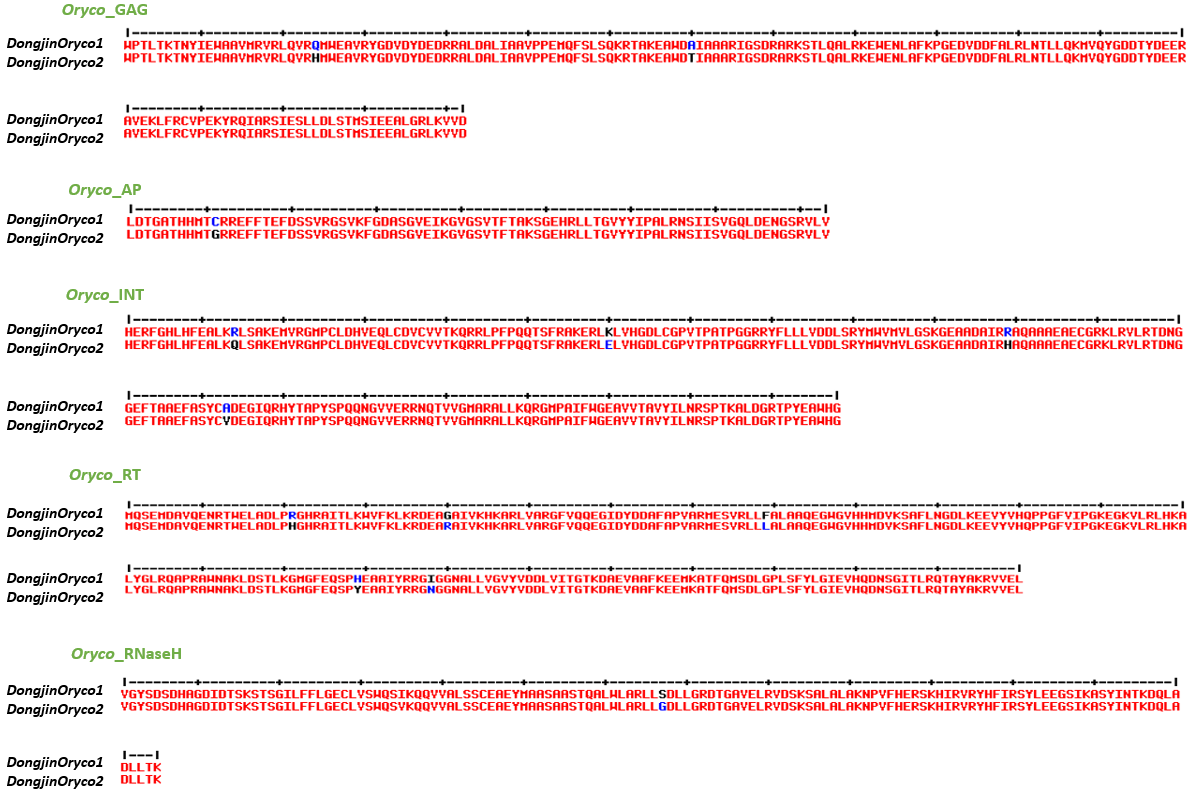


**Fig S7.** Multiple sequence alignment of the internal domain sequences of candidate active LTR-RTs belonging to the *Oryco* family. Red represents the same amino acid sequence, whereas black and blue indicate amino acid sequence variations.


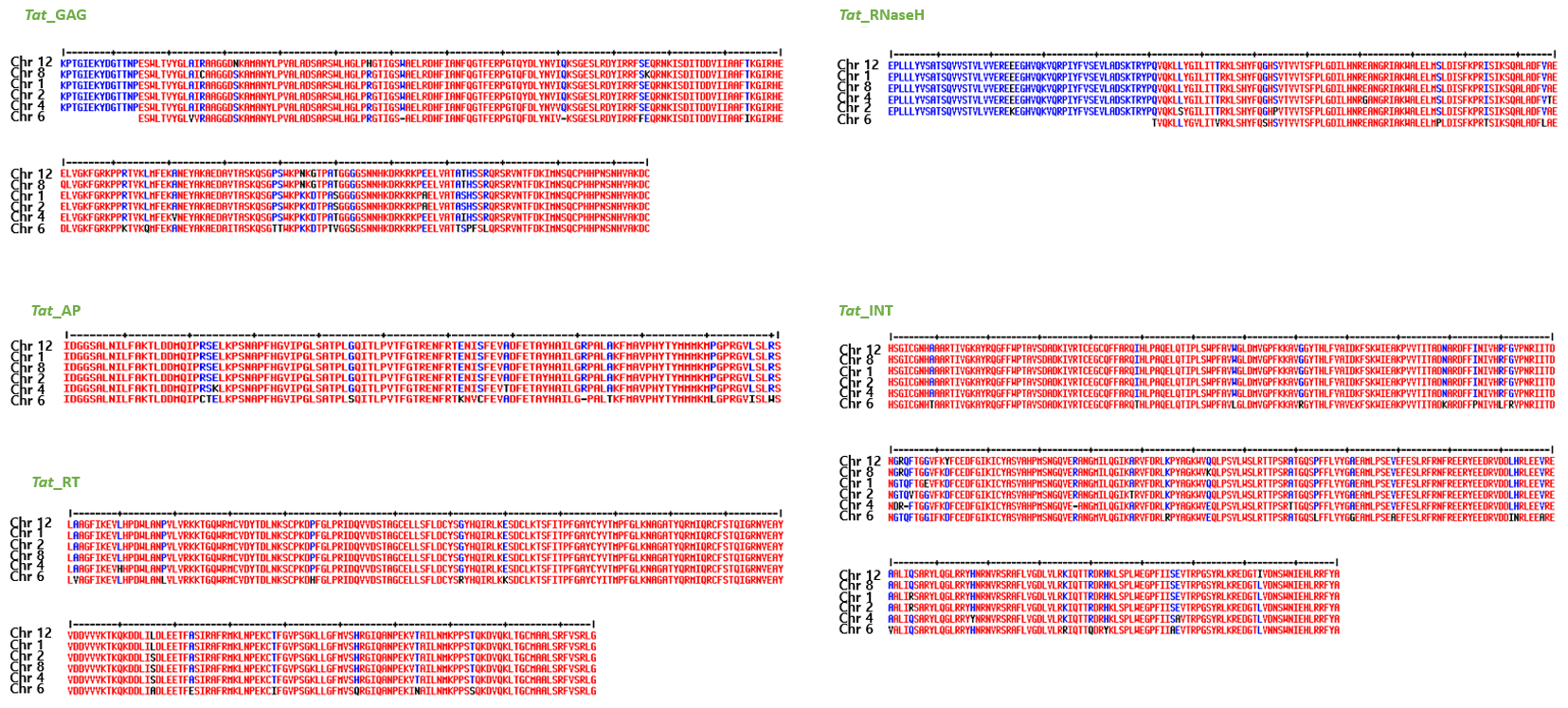
 **Fig S8.** Multiple sequence alignment of the internal domain sequences of LTR-RTs belonging to the *Tat* family with slightly increasing transpositional potential. Red represents the same amino acid sequence, whereas black and blue indicate amino acid sequence variations.


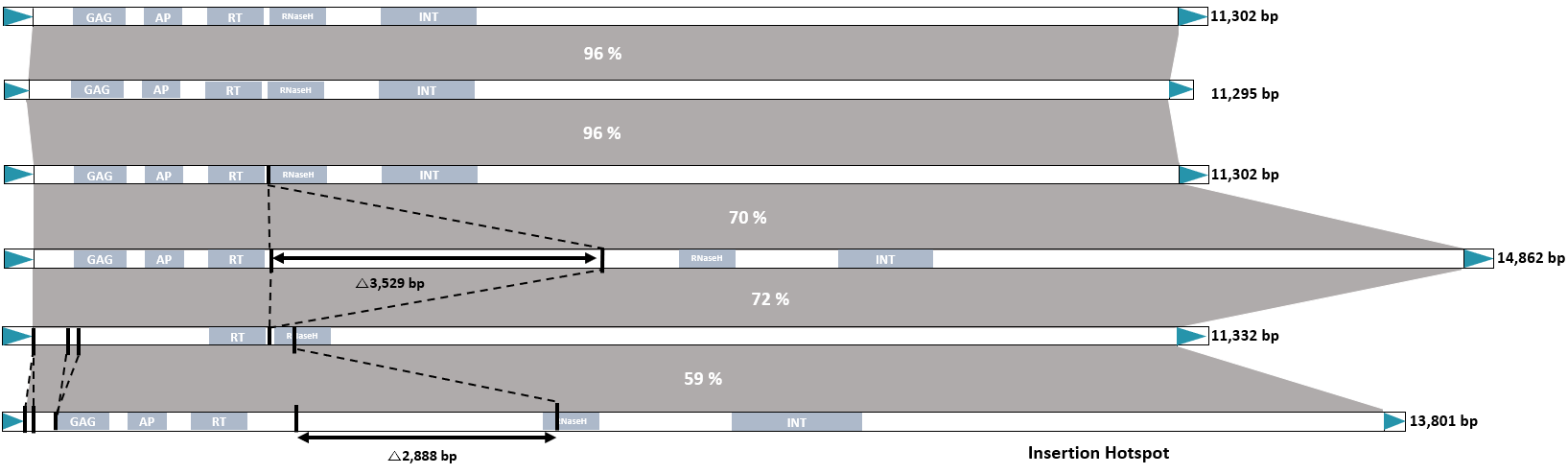


**Fig S9.** Structural characteristics of six *Tat* LTR-RTs with slightly increasing transpositional potential in the Dongjin variety. Green triangles indicate LTR regions. Gray boxes indicate the internal domains of the LTR-RTs: GAG: capsid protein, AP: aspartic protease, INT: integrase, RT: reverse-transcriptase. Gray panels represent the sequence similarity for the internal regions among six Tat family members. Indel variations for each family member are indicated.
